# Molecular Genetics of Microcephaly Primary Hereditary: An Overview

**DOI:** 10.3390/brainsci11050581

**Published:** 2021-04-30

**Authors:** Nikistratos Siskos, Electra Stylianopoulou, Georgios Skavdis, Maria E. Grigoriou

**Affiliations:** Department of Molecular Biology & Genetics, Democritus University of Thrace, 68100 Alexandroupolis, Greece; nsiskos@mbg.duth.gr (N.S.); ilstylian@mbg.duth.gr (E.S.); gskavdis@mbg.duth.gr (G.S.)

**Keywords:** microcephaly, MCPH, *MCPH1–MCPH27*, molecular genetics, cell cycle

## Abstract

MicroCephaly Primary Hereditary (MCPH) is a rare congenital neurodevelopmental disorder characterized by a significant reduction of the occipitofrontal head circumference and mild to moderate mental disability. Patients have small brains, though with overall normal architecture; therefore, studying MCPH can reveal not only the pathological mechanisms leading to this condition, but also the mechanisms operating during normal development. MCPH is genetically heterogeneous, with 27 genes listed so far in the Online Mendelian Inheritance in Man (OMIM) database. In this review, we discuss the role of MCPH proteins and delineate the molecular mechanisms and common pathways in which they participate.

## 1. Introduction

Microcephaly, from the Greek word μικροκεφαλία (mikrokephalia), meaning small head, is a term used to describe a cranium with reduction of the occipitofrontal head circumference equal, or more that teo standard deviations below the mean for age, gender, and ethnicity [1,2,3,4,5,6]. Microcephaly usually reflects a small brain volume; it is presented either as an isolated finding (non-syndromic) or with additional features, such as dysostoses and short stature (for example Seckel syndrome, Meier-Gorlin syndrome), radiosensitivity, and chromosome breakage (for example, Bloom syndrome), or diabetes (for example, Wolcott Rallison syndrome) [1,3,4]. The underlying etiology of microcephaly varies; it can be environmental, resulting, for instance, from exposure to toxic substances, or genetic [2,4]. Currently, more than 900 Online Mendelian Inheritance in Man (OMIM) phenotype entries, and approximately 800 genes with variable expressivity linked with microcephaly have been reported [1]. According to the time of diagnosis, microcephaly is classified into primary (congenital) if present at birth, or secondary, if it develops postnatally, as progressive atrophy of an initially normal brain [1,2]. Of particular interest within the genetic non-syndromic microcephaly is autosomal recessive primary microcephaly (MCPH; MicroCephaly Primary Hereditary). MCPH is a rare disease (1:10,000 to 1:250,000 live births) most frequently diagnosed in populations with consanguineous marriages [5,7,8,9,10]. The brain of MCPH patients is small, its architecture is largely normal, yet with simplified neocortical gyration [1,3,4,5,6,7]. Additionally, in some cases radiological studies reveal further abnormalities, for instance *corpus callosum* agenesis or hypoplasia, enlarged lateral ventricles, and cerebellar or brain stem hypoplasia [9,10,11,12,13,14,15,16]. Patients with MCPH usually have intellectual disability—mild to moderate retardation—their ability, however, is stable, and they can manage with daily living and safety, as well as perform simple reading and writing skills [5,15,16]. In the past 5 years, primary hereditary microcephalies inherited in an autosomal dominant manner, with clinical features as those described above, are included in the MCPH group; therefore, they are also included in the present review. MCPH is a neurodevelopmental disease with striking genetic heterogeneity; mutations in 27 genes have implicated so far, unveiling complex developmental processes that regulate mammalian brain size.

## 2. The Cellular Basis of MPCH

MCPH is the result of the reduction in the number of neurons in the brain. Brain neurons are derived from the neuroepithelial cells (NE) of the pseudostratified epithelium of the neural tube [17,18]. These cells, early in the development of the nervous system, undergo multiple rounds of symmetric divisions that expand the progenitor pool [17,18,19,20]. At the onset of neurogenesis, NE cells start expressing glial markers, adopt a radial glial (RG) cell fate and undergo multiple rounds of asymmetric cell divisions, in which they self-renew and generate neurons [19,20]. As neurogenesis continues, RG cells give rise also to intermediate progenitor (IP) cells, which undergo a limited number of symmetric cell divisions generating either transit-amplifying cells or neurons [18]. The small size of the brain observed in MCPH patients may arise from changes in the relative rates of symmetric and asymmetric divisions, or in the differentiation of the neuronal cells. These, in turn, may result from cell cycle dysregulation, i.e., from defects in timing and progression of mitosis or cytokinesis, in DNA replication and repair, or in maintenance of genome stability. In addition, the severity of microcephaly may, to a large extent, depend on the stage at which it arises; for instance, defects in NE self-proliferation may present with more severe phenotypes compared to cell cycle dysregulation in IP cells. Accordingly, as shown in Table 1, the 27 genes that have been so far linked to MCPH encode proteins that are key regulators of one or more molecular pathways that control the above-mentioned cellular processes.

Centrosomes are cellular structures consisting of a mature mother centriole and a less mature daughter centriole and act as platforms for multiple cellular functions [21]. From phase G1 to G2 centrosomes functions as microtubule-organizing centers (MTOC), during mitosis they mature into the spindle poles, and upon cell differentiation or cell cycle arrest, by elongating microtubules they transform into the basal bodies of the *cilia* [21]. Defects in centrosome biogenesis or maturation impair cell cycle progression and cell division, leading to aneuploidy, cell cycle arrest, and/or cell death [21]; in neural progenitors, these defects would result in MCPH. As already mentioned, centrosomes participate in the assembly of the mitotic spindle, which is responsible for the accurate segregation of chromosomes [22]. Three types of spindle microtubules (MTs) have been characterized within the spindle; astral MTs connect centrosomes to the cell cortex and direct the plane of division by positioning the spindle, kinetochore MTs capture condensed chromosomes, and connect them with the centrosome, and interpolar MTs interconnect the spindle poles driving the separation of the two chromosomes [22]. Defects in the organization and function of MTs, may also lead to MCPH, as mutations in proteins associated, for instance with astral MTs, which are essential for the positioning of the cleavage furrow, may affect the proportion of symmetric to asymmetric divisions of RGs. In addition to centrosome and spindle dynamics, DNA dynamics have key role in cell cycle progression [23]. During replication, cohesins associate with DNA to form the chromatids, which condense during prophase, pair in metaphase and finally segregate into the presumptive daughter cells [23]. Cellular DNA is constantly monitored for damage throughout the cell cycle; once damage is detected, repair is needed before progressing into the next stage of the cell cycle [23]. As a result, mutations in genes involved in replication, chromosomal condensation or segregation, or damage repair may affect for example the timing of the cell cycle causing delays [22], which eventually lead to reduced numbers of NEs, RGs or IPs, and hence, to MCPH.

## 3. *MCPH* Genes

*MCPH1*, the first *MCPH* gene, was identified nearly 20 years ago [24]; however, it is during the last decade with the rapid progress in next generation sequencing technology that most of the MCPH genes and variants have been characterized [7,8,9,24]. As shown in Table 1, nearly all MCPH genes identified so far are implicated in one or more of the processes involved in the cell cycle, having usually distinct roles at different phases and/or in different molecular pathways.

### 3.1. ASPM (MCPH5)

Mutations in *ASPM* are the most frequent cause of MCPH, accounting for 68.6% of the cases in consanguineous patients [10,25]. *ASPM* encodes a protein with two calponin homology domains, a microtubule-binding domain and multiple IQ calmodulin-binding motifs [26,27,28]. During interphase, ASPM is localized at the centriole, while in mitosis it is detected at the spindle poles [26,27,29]. In mouse telencephalic neural progenitor cells, in the absence of functional ASPM spindle positioning is altered and divisions shift from symmetrical to asymmetrical instead of, however, cell cycle arrest [30], suggesting that ASPM at the spindle poles acts by maintaining symmetric divisions [30,31]. Notably, ASPM expression in the embryo is downregulated with the onset of neurogenic divisions [30,31]. Moreover, mice expressing *ASPM* variants, similar to those identified in patients with MCPH, exhibit mild microcephaly, and can be rescued by the introduction of a transgene encoding the wild type human protein [31]. Mutations in the *Drosophila* homolog of *ASPM* cause severe reduction of brain size due to defects in mitosis and increased apoptosis in the developing neuroepithelium [32]; similarly, knockdown of the zebrafish homolog leads to head size reduction [33]. More recently, ASPM has been implicated in centriole biogenesis [27,34]. During interphase, ASPM localizes at the proximal end of the mother centriole as component of a protein complex that is essential for centriole duplication; in mouse brain, lack of functional ASPM impairs centriole duplication leading to a reduction in the number of centrosomes and *cilia* in the cells [27].

### 3.2. WDR62 (MCPH2)

*WDR62* encodes a protein with a WD40 domain, a JNK (c-JunN-terminal Kinase) docking domain, and a MKK7 binding domain. Mutations in *WDR62* are the second most frequent cause of MCPH, accounting for approximately 10% of the cases [10,35,36]. During mitosis, WDR62 is localized at the spindle poles [35]. Following bipolar spindle formation, WDR62 has a key role in stabilizing the spindle poles; in the absence of WDR62, the distribution of PCM components such as γ-tubulin or pericentrin is altered [37]. Moreover, during mitosis, WDR62 is a substrate of JNK active at the centrosome; phosphorylated WDR62 is required for the organization of the mitotic spindle [37,38]. ASPM interacts with WDR62; this interaction is mediated by CEP63, a protein linked to Seckel syndrome, characterized by severe microcephaly with intellectual disability and short stature [34]. Knockdown of either *Wdr62* or *JNK* using shRNA in neural progenitor cells of the rat telencephalon result in spindle misorientation, asymmetric divisions and, thus, in premature differentiation to neurons [39]. These defects are rescued by expressing human WDR62 protein but not MCPH-associated WDR62 mutant proteins [39]. Moreover, in mice, neural progenitor cells lacking functional WDR62 exhibit delayed mitotic progression leading to cell death and therefore, to small brain size [40]. In zebrafish, knockdown of WDR62 using morpholinos impairs mitotic progression causing increased cell death, resulting thus, in significant reduction in head size [41]. During interphase, WDR62 localizes at the proximal end of the mother centriole and its function is essential for the recruitment of proteins in a complex required for centriole biogenesis; in the mouse brain absence of WDR62 causes reduction of the number of centrosomes and *cilia* due to defects in centrosome biogenesis [34,42].

### 3.3. MCPH1 (MCPH1)

*MCPH1*, the first *MCPH* gene identified, encodes Microcephalin 1, a protein with three BCRT (BRCA1 C-terminus) domains; the N-terminal BRCT domain interacts with the SWI–SNF complex, while the C-terminal BRCT domains mediate MCPH1 interactions with phosphorylated peptides [43]. Mutations in *MCPH1* are the third most frequent cause of MCPH accounting for approximately 8% of the cases [9]. Microcephalin 1 has various roles in the cell cycle. It is required for the localization of the checkpoint kinase1 (Chk1); in the absence of Microcephalin 1, untimely entry to mitosis is observed leading to defects in the organization of the spindle and/or chromosome misalignment associated with premature chromosome condensation [43,44,45,46]. Microcephalin 1 acts as a transcriptional repressor of hTERT and interacts with the anaphase-promoting complex via Cdc27 linking transcription with cell cycle progression [43,45,47]. In addition, it interacts with histone H2AX and is involved in the DNA damage repair mechanisms acting as tumor suppressor [43,45,48,49,50]. In mice, knockout of *Mcph1* leads to defects in chromosome condensation, deficiencies in DNA repair, spindle misorientation and, eventually, to a reduction of the brain size mimicking the human disease [51]. In the neuroprogenitors of these mice, the lack of Microcephalin 1 leads to a shift from symmetric to asymmetric divisions, reducing the neuroprogenitor pool and, as a result, the number of neurons in the brain [51]. In *Drosophila*, mutations in the homolog of *MCPH1* are embryonic lethal with mutant embryos exhibiting abnormal centrosomes, asynchronous nuclear and centrosomal cycles, defective spindle and premature chromosome condensation, leading to mitotic arrest [52,53].

### 3.4. CDK5RAP2 (MCPH3)

*CDK5RAP2* gene encodes a protein-regulator of CDK5 activity that interacts with γ-tubulin promoting microtubule nucleation in the pericentriolar material of the centrosome [54,55,56]. Recruitment of CDK5RAP2 depends on pericentrin and WDR62 [34,56]. During mitosis, CDK5RAP2 and pericentrin engage CEP192 to the spindle poles; this interaction can direct spindle formation in the absence of centriole [57,58]. Moreover, CDK5RAP2 acting as transcription activator, regulates the expression of *BUBR1* and *MAD2*, two genes implicated in the control of spindle checkpoints [58]. CDK5RAP2 is expressed in neural progenitor cells; mutant mice lacking CDK5RAP2 exhibit mild microcephaly [59,60,61]. Furthermore, at the cellular level, in the neuroprogenitors of these mice, premature cell cycle exit and increased apoptosis are observed due to the disrupted structure of the centrosomes and the presence of multipolar spindles [59,60,61].

### 3.5. CASC5 (KNL1, MCPH4)

*CASC5* encodes a scaffold protein of the kinetochore that is essential for the attachment of chromatin to the mitotic apparatus and interacts with BUB1 and BUBR1 to control the spindle assembly checkpoint [62,63]. Reduction in CASC5 levels leads to chromosome misalignment [63]. In cells derived from MCPH patients, CASC5 localizes at the metaphase plane, as well as in the cytoplasm; in these cells, the percentage of mitotic and apoptotic cells is high, probably due to mitotic delay and DNA damage [64].

### 3.6. CENPJ (SAS-4, CPAP, MCPH6)

*CENPJ* encodes a protein that consists of phosphorylation domains, five coiled-coil domains and a C-terminal domain with 21 G-box repeats and a leucine zipper motif [65]. CENPJ is localized in the centrosome and is required for centriole integrity and duplication [27,65]. It binds MTs and interacts with several other centrosomal proteins implicated in MCPH [27,66,67]. CENPJ is also involved in ciliogenesis; lack of functional CENPJ in neural progenitor cells results in delayed *cilium* disassembly and cell-cycle re-entry, promoting, thus, differentiation [68]. The phenotype of mice with targeted inactivation of *Cenpj* exhibit several of the clinical findings observed in patients suffering not only from MCPH, but also from Seckel syndrome [69,70].

### 3.7. STIL (MCPH7)

*STIL* is an oncogene encoding a protein that does not share any known structural motifs with other proteins. It is localized to the centrioles with a role in centriole biogenesis and spindle pole positioning [71,72]. STIL interacts with several other centrosomal proteins implicated in MCPH [27,66,67,71]. Upregulation of *STIL* leads to the generation of supernumerary nascent centrioles localized close to the parental centriole, a phenotype resembling the effect of the overexpression of two other genes, namely *SASS-6* and *PLK4* [73]. Moreover, STIL is required for proper positioning of the mitotic spindle and, hence, mitotic progression [71,74]. Knockout of *STIL* in mice or its ortholog *sil* in zebrafish, leads to embryonic lethality—cells lack centrioles and *cilia*; however, the cause of lethality is not clear and may be related to other roles of STIL, for instance, in hedgehog signaling [74,75]. Interestingly, STIL has recently been shown to have a role in the generation and regeneration of dopaminergic neurons [76].

### 3.8. CEP135 (MCPH8)

*CEP135* encodes a protein with a coiled-coil structure that in vitro interacts with tubulin, protofilaments, and microtubules [77,78]. It is localized at the centrosome and has a role in centriole biogenesis and integrity, interacting during different phases of the cell cycle with several proteins, including the products of *MCPH* genes, *SASS6* and *CENPJ* [79,80,81]. In both vertebrates and invertebrates, absence of CEP135 results in various structural defects of the centrioles [82,83].

### 3.9. CEP152 (MCPH9)

*CEP152* encodes a protein with several coiled-coil domains that is localized at the centrosome [84,85,86]. Mutations in *CEP152* are a rare cause of MCPH; however, they are a frequent cause of Seckel syndrome [16,84,85]. CEP152 interacts with several proteins during the cell cycle; interestingly, CEP152 interacts with another protein linked with Seckel syndrome, namely CEP63, to cooperatively generate an initial complex that self-assembles into a higher-order cylindrical structure that recruits downstream components for centriole duplication [87]. In addition, CEP152 interacts with CINP, a protein implicated in DNA damage response and genome maintenance; thus, it is also involved in the regulation of cell cycle checkpoints [88]. Loss of CEP152 results in the formation of monopolar spindles, chromosomal instability and, subsequently, mitotic defects [89].

### 3.10. ZNF335 (MCPH10)

*ZNF335* encodes a zinc finger protein, of the H3K4 methyltransferase complex, localized in the nucleus, with roles in chromatin remodeling and transcriptional regulation [90,91]. *ZNF335* is expressed in neural progenitor cells and prevents early cell cycle exit by regulating the levels of REST/NRSF, a key regulator of the proliferation and neuronal differentiation [91]. Mutations of *ZNF335* cause severe microcephaly associated with neuronal disorganization and small brain size, therefore its characterization as *MCPH* gene has been questioned [7]. In mice, conditional knockout of *Znf335* in the nervous system results in severely reduced cortical size, premature differentiation, and impaired neuronal morphogenesis [91].

### 3.11. PHC1 (MCPH11)

*PHC1* encodes a component of the Polycomb complex and is localized in the nucleus [92]. PHC1 interacts with H2A and is required for its ubiquitination; cells derived from MCPH11 patients are characterized by defects in DNA repair, at baseline and following irradiation, implicating PHC1 in DNA damage repair [92,93]. Additionally, PHC1 interacts with Geminin, a protein that inhibits DNA replication; in cells that lack PHC1 geminin degradation is impaired, leading to abnormal cell cycle [93]. In *Pch1,* knockout mice anteroposterior patterning as well as heart and skeleton development are affected [94]. Furthermore, *Pch1* is required for the activity of hematopoietic stem cells in a gene dosage-dependent manner [95,96].

### 3.12. CDK6 (MCPH12)

*CDK6* is a member of the CDK family that includes proteins that have key role in cell cycle progression in G1 and S phase. During interphase, CDK6 is localized in the cytosol and in the nucleus; however, it is also found at the centrosomes and at the spindle poles [97]. Patient-derived cells exhibit reduced cell proliferation, while defects in the organization of the MTs and of the mitotic spindles, as well as supernumerary centrosomes are observed [97]. Mice lacking Cdk6 develop normally [98]; however, given that at the onset of neurogenesis in the developing mouse cortex, transcription factor Pax6 directly represses *Cdk6* increasing the length of the G1 phase [99], the human phenotype may reflect novel functions of CDK6 related to the development of the primate telencephalon [100].

### 3.13. CENPE (MCPH13)

*CENPE* encodes a kinesin-like motor protein that is expressed during the cell cycle with the highest levels at G2/M phase [101]. CENPE is required for capturing spindle MTs and attachment to the kinetochores [102,103]. Defects in the organization of spindle MTs and delayed progression of the mitosis, leading to abnormal exit from mitosis have been described in MCPH13 patient-derived cell lines as well as in medulloblastoma [101,104]; the presence of binucleate cells of unequal size suggests that, in the absence of CENPE, chromosome segregation and cytokinesis fails [101,104]. Knockout of *Cenpe* in mice is embryonic lethal, however, in conditional mutants, severe mitotic chromosome misalignment and chromosome segregation fails, in accordance with the observations in human cells [105,106].

### 3.14. SASS6 (SAS-6, MCPH14)

*SASS6* encodes a protein with a coiled-coil domain that is expressed in the cells from G2 until mitosis [107,108]. At the onset of centriole duplication, SASS6 is recruited to the centriole and acts as a scaffolding component and during centriole biogenesis it interacts with several proteins [80,86,87,109,110]; overexpression of SASS6 leads to centrosome duplication [111,112].

### 3.15. MFSD2A (MCPH15)

*MFSD2A* encodes a fatty acid transporter critical for blood–brain–barrier (BBB) function that is expressed in the endothelium of the BBB and in neural stem cells [113]. MFSD2A transports molecules that are required for neurogenesis but are not synthesized in the brain, such as docosahexaenoic acid (DHA) [113,114,115,116]. Interestingly, mice lacking functional Mfsd2A in the BBB develop initially DHA deficiency in the brain and then mild microcephaly [116,117].

### 3.16. ANKLE2 (LEM4, MCPH16)

*ANKLE2* encodes a protein with ankyrin repeat and a LEM domain that is localized in the endoplasmic reticulum and the nuclear envelope and is essential for their integrity [118]. It was first characterized in *Drosophila* in a mutagenesis screen aiming to identify mutants with neurodevelopmental abnormalities [119]. Subsequently, the human homolog was used to search the exome database of the Baylor-Hopkins Center for Mendelian Genomics and an individual was identified bearing variants in *ANKLE2* [118,119,120]. In *Drosophila*, *Ankle2* is essential for the segregation of cell fate determinants during the asymmetric divisions of neuroblasts; in *Ankle2* mutants, neuroblasts self-renewal is abolished and the larvae exhibit small brains [118,119,120]. Notably, the NS4A protein of ZIKA virus targets ANKLE2 pathway causing environmental microcephaly [118].

### 3.17. CIT (MCPH17)

*CIT* encodes two major isoforms that bind to the active form of the small GTPase RhoA; CIT-K is the largest and is specifically expressed in neural progenitor cells in the developing brain, while CIT-N, which lacks the kinase domain, is expressed in post mitotic neurons [121]. Mice lacking isoform CIT-K, but expressing normal levels of CIT-N, develop microcephaly as a result of cytokinesis defects and DNA double strand breaks that activate P53 [122,123]. CIT-K is localized at the spindle poles during metaphase and later at the mid-body [121] and is required for its structural integrity as well as to maintain active RhoA [124]. Fibroblasts isolated from MCPH17 patients do not show any defects, however when patient derived iPS are differentiated to neural stem cells, cytokinesis failure followed by apoptosis is observed [125].

### 3.18. ALFY (WDFY3, MCPH18)

*ALFY* encodes a phosphatidylinositol 3-phosphate-binding protein, which acts as scaffold protein to facilitate selective autophagy-mediated removal of aggregated intracellular proteins and clearance of mitochondria via mitophagy; a dominant missense mutation of *ALFY*, has been linked to microcephaly through clearance of DVL3, a target of the canonical Wnt signaling pathway [126]. In *Drosophila*, expression of the human mutant allele leads to small brain, recapitulating the human phenotype [126]. In the developing mouse brain, ALFY is expressed in the neocortex regulating the proliferation of neural progenitor cells; lack of *ALFY* product results in the expansion of the radial glial cell population as the number of asymmetric divisions is reduced [127]. Notably, several recessive mutations of *ALFY* have been linked with macrocephaly underlying the opposing effects that the expression levels of this gene have on brain development [128].

### 3.19. COPB2 (MCPH19)

*COPB2* encodes a subunit of the Golgi coatomer complex required for the retrograde trafficking from the Golgi complex to the endoplasmic reticulum [129]. Hypomorph mutations of *COPB2* have been linked to microcephaly; however, mice homozygous for patient variants are normal, probably reflecting species differences [130]. Recently, COPB2 silencing has been shown to inhibit cell proliferation, inducing apoptosis via the JNK/c-jun pathway in a cancer cell line [131].

### 3.20. KIF14 (MCPH20)

*KIF14* encodes a member of the kinesin-3 motor family that is localized at the mitotic spindle, at the spindle midzone to sustain its structure and at the midbody where it acts together with CIT-K to promote cytokinesis [132]. Knockout mice lacking *Kif14* product exhibit severe microcephaly [133]; recently, exome-sequencing analysis identified microcephaly-causing variants of KIF14 [134]. In mitotic cells derived from MCPH20 patients, neither KIF14 nor CIT-K were detected at the midbody resulting in cytokinesis failure [134].

### 3.21. NCAPD2 (CNAP1, MCPH21)

*NCAPD2* encodes a subunit of condensin I required for the compaction of chromosomes following the breakdown of the nuclear envelope [135]. NCAPD2 contains a nuclear localization signal required also for chromosome targeting and a HEAT repeat, a repetitive array of amphiphilic α-helices used in protein-protein interactions [136]. The interaction between NCAPD2 and the phosphorylated histone H3 targets condensin I on the chromosomes [137]. Lack of functional NCAPD2 affects mitotic chromosome assembly and resolution of sister chromatids [138]. Moreover, NCAPD2 interacts with rootletin, regulating centrosome cohesion and reducing DNA damage caused during centriole splitting [138]. Recently, hypomorphic *NCAPD2* mutations leading to decatenation defects that impair chromosome segregation during mitosis have been linked with microcephaly [139,140].

### 3.22. NCAPD3 (MCPH22)

*NCAPD3* encodes a subunit of condensin II, which is required for the early stage of axial shortening during prophase [135]. NCAPD3 contains a HEAT repeat; phosphorylation of NCAPD3 by the mitotic kinase Cdk-1 is required to timely initiate chromosome condensation during prophase [141]. Hypomorphic *NCAPD3* mutations leading to decatenation defects that impair chromosome segregation at mitosis have been linked with microcephaly [139]. Fibroblasts derived from patients with *NCAPD3* mutations exhibit impaired chromosome segregation [139,142].

### 3.23. NCAPH (MCPH23)

*NCAPH* encodes a subunit of condensin I member of the kleisin family of proteins that binds to ATP-binding cassette (ABC)-transporter-like ATPase domains [135]. NCAPH binds directly to DNA and is required for the stable interaction of the condensin complexes with the chromosomes [143]. One patient homozygous for a missense mutation in *NCAPH* has been reported [139]; patient-derived fibroblasts showed impaired chromosome segregation followed by abnormal recovery from condensation [139].

### 3.24. NUP37 (MCPH24)

*NUP37* encodes one of the components of the nuclear pore subcomplex Nup107-160 required for the assembly of a functional nuclear pore complex [144]. During mitosis, Nup107-160 is essential for the nucleation of MTs on mitotic kinetochores and spindle assembly [144]. So far, one missense mutation in *NUP37* has been described by exome sequencing in a family; patient fibroblasts have fewer nuclear pore complexes, altered structure of the nuclear envelope and decreased cellular proliferation rate [145].

### 3.25. MAP11 (TRAPPC14, C7orf43, MCPH25)

*MAP11* encodes a component of the vesicle tethering complex TRAPP II that functions in late Golgi trafficking as a membrane tether [146]. MAP11 is dispensable for TRAPPII activity in Golgi trafficking, but it is essential for the preciliary vesicle trafficking to the mother centriole during ciliogenesis [146]. Recently, a nonsense mutation in *MAP11* was shown to cause microcephaly; in addition, knockout of the gene in zebrafish resulted in microcephaly [147]. In SH-SY5Y cells, MAP11 associates with α-tubulin; this association is observed both during mitosis at the spindle as well as later, in cytokinesis at the midbody [147]. Knockdown of *MAP11* in SH-SY5Y cells reduces cell proliferation [147].

### 3.26. LMNB1 (MCPH26) and LMNB2 (MCPH27)

*LMNB1* and *LMNB2* encode two related components of the nuclear lamina with coiled-coil domains that form filaments and interact with various proteins [148]. In addition, LMNB1 and LMNB2 associate with the mitotic spindle; dominant negative mutant proteins that disrupt the organization of the filaments, impair the formation of the mitotic spindle [149]. Recently, dominant mutations in *LMNB1* and *LMNB2* have been shown to cause primary microcephaly [150,151]; the analysis of these variants in *HeLa* cells revealed defects in the formation of the nuclear envelope [150]. Interestingly, mice lacking Lmnb1 or Lmnb2 exhibit neuronal migration defects as well as reduced number of neuronal cells in the cerebral cortex [152,153].

## 4. Cellular Processes and Molecular Pathways in MCPH

In the past 10 years, advances in next generation sequencing technologies and bioinformatics have accelerated the identification of novel genes and variants associated with MCPH (Table 1). Interestingly, in several cases, only a single patient (*NCPAD2*, *NCAPH*), or very few patients (*MAP11*, *NUP37*, *COPB2*, *CENPE*) with variants in one *MCPH* gene have been identified, underlining the effectiveness of the current approaches in rare disease diagnosis [154]. The discovery and analysis of novel *MCPH* genes and variants is of paramount importance for the understanding not only the pathological mechanisms leading to the disease, but also the mechanisms underlying normal human brain development.

The majority of MCPH cases (approximately 80%) are caused by mutations in *ASPM* or *WDR62*; both proteins are localized at the centrosome and the spindle poles indicating that brain size is particularly vulnerable to mutations that affect these structures. Interestingly, at the centrosome, not only the localization but also the roles of ASPM and WDR62 are closely linked; ASPM interacts with WDR62 and they both participate along with CEP63 in a protein complex that is required for centriole duplication [27,155]. Moreover, the products of eight other *MCPH* genes have key roles in centriole biogenesis. More specifically, at the proximal end of the parental centriole, WDR62 recruits CEP63; in the absence of WDR62, centrosomal localization of CEP63 is impaired [27]. The next step involves the recruitment of ASPM by WDR62 and of CEP152 by CEP63 [27,155]. CEP152–CEP63 interaction triggers the engagement of the kinase Plk-4, which autophosphorylates and subsequently phosphorylates STIL, which in turn recruits SASS6 [155,156,157,158,159]. SASS6 and STIL oligomerize and form the core of the daughter centriole, engaging two other MCPH proteins, CEP135 and CENPJ, essential for microtubule nucleation and lengthening of the centriole [86,108,155,156,160,161]. ASPM and WDR62 are also localized at the pericentriolar material where other proteins, for instance CDK5RAP2 and pericentrin, are recruited, and microtubules start assembling, as the daughter centriole is forming [30,162,163]. Additionally, CDK5RAP2 is essential for centriole and centrosome cohesion as in its absence, centriole splitting is detected resulting in supernumerary centrosomes [57]. Microcephalin 1 recruits Chk1 to the centrosome where it acts to ensure the coupling of the centrosome cycle with mitosis [44].

One striking feature of the majority of the MCPH proteins is that they have overlapping roles in cellular functions and usually operate in more than one pathways that influence the cell cycle; hence, several *MCPH* genes discussed above, have also key roles in spindle structure and function. ASPM is involved in spindle assembly and orientation recruiting CIT-K and dynein–dynactin at the poles, WDR62 has a key role in stabilizing the spindle poles through protein–protein interactions, and CDK5RAP2 supports γ-tubulin induced microtubule nucleation in the pericentriolar material scaffold [29,35,37,38,58,144,164,165]. Furthermore, other MCPH proteins operate at the spindle; NUP37 as part of the Nup107-160 complex promotes its assembly through microtubule nucleation while CDK6 also functions at the spindle poles [144,166]. Moreover, LMNB1 and LMNB2 are essential for mitotic spindle assembly by organizing into a matrix-like network that depends on RanGTP [149]. CASC5 forms the scaffold of the kinetochore for the microtubule attachment to the centromere, controlling also the spindle assembly checkpoint, while CENPE is required for the attachment of the spindle microtubules to the kinetochore; both CENPE and CASC5 are also implicated in the spindle assembly checkpoint [62,63,103,167,168]. During anaphase, ASPM interacts with CIT-K and, along with CDK5RAP2 and CASC5, they localize at the midbody; KIF14 is engaged by CIT-K and along with MAP11 promote cytokinesis [27,132,169,170,171].

Besides centriole biogenesis and spindle dynamics during the cell cycle, several MCPH proteins are implicated in pathways related with DNA dynamics; the recently identified as MCPH proteins, NCPAD2, NCPAD3, and NCPAH are all involved in chromosome condensation as components of the condensin complexes [135]. Moreover, Microcephalin 1 acts as regulator of chromosome condensation that inhibits condensin II, interacting both with NCAPD3 and NCAPG2 subunits [172]. Microcephalin 1 and PHC1 are also implicated in DNA damage repair; PCH1 and ZNF335 have a role in chromatin remodeling and transcription [91,92]. Finally, the (de)phosphorylation of nuclear envelope proteins by ANKLE2 regulates the affinity between the nuclear envelope and the chromatin during the cell cycle [154].

Despite the progress that has been made in MCPH molecular genetics, only in the 50% of the patients has the gene causing mutation been identified; these observations along with the complex genetic interactions described above raise the possibility of an oligogenic model of inheritance with variable expressivity and/or incomplete penetrance. Notably, while *Aspm−/−* mice exhibit mild reduction in brain volume, this phenotype is enhanced if the animal is heterozygous for a loss-of-function mutation in *Wdr62*; furthermore, lack of both Aspm and Wdr62 leads to embryonic lethality [27]; similar results were obtained in zebrafish [173]. In-depth analysis of high-throughput DNA sequencing data revealed that patients with MCPH carry a significant burden of variants in 75 genes (*MCPH* genes included) and identified cases of digenic inheritance [173]. These results pave the way for new approaches in the diagnosis of MCPH by exploiting the molecular pathways that have been identified.

In this review, we focused on the genes involved in non-syndromic primary microcephaly; therefore, genes involved in syndromes associated with primary microcephaly were not discussed. Nevertheless, it is worth noting that CENPJ and CEP152 have been also linked with Seckel syndrome (Table 1), while CEP63, which has been primarily implicated in Seckel syndrome, is also linked to MCPH. Moreover, CENPE has been also linked to microcephalic primordial dwarfism [70,85,101,157]. Given that microcephalies are rare diseases with digenic or oligogenic modes of inheritance in some cases, it is possible that several genes will finally be linked with more than one type of microcephaly, highlighting, thus, the common molecular pathways and pathological mechanisms involved in a spectrum of disorders, including MCPH and related conditions.

## Figures and Tables

**Table 1:** *MCPH* genes (February 2021).

Disorder	OMIM	Chromosomal Location	Gene	Protein	Mode of Inheritance	Subcellular Localization	Cellular Process (es)
MCPH1	607117	*8p23.1*	*MCPH1*	Microcephalin 1 (BRCT-repeat inhibitor of hTERT, MCPH1)	Autosomal recessive	Nucleus Centrosome	DNA damage Chromatin condensation Coupling centrosome cycle to mitosis
MCPH2	613583	*19q13.12*	*WDR62 (MCPH2)*	WDR62 (WD Repeat-containing protein 62, MCPH2)	Autosomal recessive	Centrosome Spindle poles	Centriole biogenesis Spindle assembly and orientation
MCPH3	608201	*9q33.2*	*CDK5RAP2 (MCPH3)*	CDK5RAP2 (CDK5 Regulatory subunit Associated Protein 2, MCPH3)	Autosomal recessive	Pericentriolar material of the centrosome Nucleus	Centriole biogenesis Control of spindle checkpoints Cytokinesis
MCPH4	609173	*15q15.1*	*CASC5 (KNL1, MCPH4)*	CASC5 (CAncer Susceptibility Candidate 5, KNL1, kinetochore scaffold 1, MCPH4)	Autosomal recessive	Kinetochore	Attachment of chromatin to the mitotic apparatus Control of spindle checkpoint
MCPH5	605481	*1q31.3*	*ASPM (MCPH5)*	ASPM (Abnormal SPindle Microtubule assembly, MCPH5	Autosomal recessive	Nucleus Centrosome Midbody	Centriole biogenesis Spindle assembly and orientation Cytokinesis
MCPH6	609279	*13q12.12-q12.13*	*CENPJ (SAS-4, CPAP, MCPH6)*	CENPJ (CEntromere Protein J, SAS-4, CPAP, MCPH6)	Autosomal recessive	Centrosome	Centriole biogenesis
MCPH7	181590	*1p33*	*STIL (MCPH7)*	STIL (SCL/TAL1 Interrupting Locus, MCPH7)	Autosomal recessive	Centrosome	Centriole biogenesis Spindle assembly and positioning
MCPH8	611423	*4q12*	*CEP135 (MCPH8)*	CEP135 (CEntrosomal Protein 135, MCPH8)	Autosomal recessive	Centrosome	Centriole biogenesis
MCPH9	613529	*15q21.1*	*CEP152 (MCPH9)*	CEP152 (CEntrosomal Protein 152, MCPH9)	Autosomal recessive	Centrosome	Centriole biogenesis
MCPH10	610827	*20q13.12*	*ZNF335 (MCPH10)*	CEP152 (CEntrosomal Protein 152, MCPH9)	Autosomal recessive	Nucleus	Transcription Chromatin remodeling
MCPH11	602978	*12p13.31*	*PHC1 (MCPH11)*	PHC1 (PolyHomeotiC like 1, MCPH11)	Autosomal recessive	Nucleus	Transcription Chromatin remodeling
MCPH12	603368	*7q21.2*	*CDK6 (MCPH12)*	CDK6 (Cyclin Dependent Kinase 6, MCPH12)	Autosomal recessive	Cytosol Nucleus Spindle poles Centrosome	Cell cycle regulation
MCPH13	117143	*4q24*	*CENPE (MCPH13)*	CENPE (CENtromere associated Protein E, MCPH13)	Autosomal recessive	Kinetochore	Kinetochore attachment Control of spindle checkpoint
MCPH14	609321	*1p21.2*	*SASS6 (SAS6, MCPH14)*	SASS6 (Spindle ASSembly abnormal protein 6 homolog, MCPH14)	Autosomal recessive	Centrosome	Centriole biogenesis
MCPH15	614397	*1p34.2*	*MFSD2A (MCPH15)*	MFSD2A (Major Facilitator Superfamily Domain containing 2A, MCPH15)	Autosomal recessive	Plasma membrane	Metabolism
MCPH16	616062	*12q24.33*	*ANKLE2 (LEM4, MCPH16)*	ANKLE2 (ANKyrin repeat and LEM domain containing protein 2, MCPH16)	Autosomal recessive	Endoplasmic reticulum Nuclear envelope	Nuclear envelope assembly/disassembly
MCPH17	605629	*12q24.23*	*CIT (MCPH17)*	CIT (CITron rho-interacting serine/threonine kinase, MCPH17)	Autosomal recessive	Spindle Midbody	Spindle assembly and orientation Cytokinesis
MCPH18	617485	*4q21.23*	*ALFY (WDFY3, MCPH19)*	ALFY (Autophagy-Linked FYVE protein, WDFY3, MCPH18)	Autosomal dominant	Cytoplasm Nucleus	Canonical Wnt pathway
MCPH19	606990	*3q23*	*COPB2 (MCPH19)*	COPB2 (COatomer Protein complex, subunit Beta 2, MCPH19)	Autosomal recessive	Non-clathrin vesicles	Vesicle trafficking Apoptosis via the JNK/c-jun pathway
MCPH20	611279	*1q32.1*	*KIF14 (MCPH20)*	KIF14 (Kinesin Family member 14, MCPH20)	Autosomal recessive	Spindle poles Spindle mid-zone Midbody	Spindle assembly Cytokinesis
MCPH21	615638	*12p13.31*	*NCAPD2 (CNAP1 MCPH21)*	NCAPD2 (Non-SMC condensin I complex Subunit D2, Centrosomal Nek2-Associated Protein 1, MCPH21)	Autosomal recessive	Nucleus Chromatin Chromosomes	Chromatin condensation
MCPH22	609276	*11q25*	*NCAPD3 (MCPH22)*	NCAPD3 (Non-SMC condensin II complex subunit D3, MCPH22)	Autosomal recessive	Nucleus Chromatin Chromosomes	Chromatin condensation
MCPH23	602332	*2q11.2*	*NCAPH (MCPH23)*	NCAPH (Non-SMC condensin I complex subunit H, MCPH23)	Autosomal recessive	Nucleus Chromatin Chromosomes	Chromatin condensation
MCPH24	609264	*12q23.2*	*NUP37 (MCPH24)*	NUP37 (NucleoPorin 37, MCPH24)	Autosomal recessive	Nuclear Pore Kinetochore	Nuclear Pore assembly Spindle assembly
MCPH25	618350	*7q22.1*	*MAP11 (TRAPPC14, C7orf43, MCPH25)*	MAP11 (Microtubule Associated Protein 11, TRAPPC14, C7orf43, MCPH25)	Autosomal recessive	Spindle Midbody Golgi	Spindle assembly Cytokinesis Golgi trafficking
MCPH26	150340	*5q23.2*	*LMNB1*	LMNB1 (LaMiN B1, MCPH26)	Autosomal dominant	Nuclear Lamina Spindle	Nuclear envelope assembly Assembly of the mitotic spindle
MCPH27	150341	*19p13.3*	*LMNB2*	LMNB2 (LaMiN B2, MCPH27)	Autosomal dominant	Nuclear Lamina Spindle	Nuclear envelope assembly Assembly of the mitotic spindle
